# External quality assessment (EQA) of *Neisseria gonorrhoeae* antimicrobial susceptibility testing in primary laboratories in Germany

**DOI:** 10.1186/s12879-020-05234-w

**Published:** 2020-07-16

**Authors:** Regina Selb, Klaus Jansen, Matthias Eckardt, Thalea Tamminga, Sandra Dudareva, Martyna Gassowski, Ingeborg Graeber, Eva Guhl, Dagmar Heuer, Susanne Buder, the GORENET EQA study group, Veronika Balau, Daniela Nagel, Anja Berger, Andreas Sing, Valerie Chapot, Jörg Steinmann, Heinrich Fischer, Siegfried Kösel, Vanessa Dreyer, Ute Tonnemacher, Andreas Groß, Hans Jochen Hagedorn, Tilo Hackel, Alexander Halfmann, Sören Becker, Christina Hess, Benjamin Blümel, Simone Korten, Hany Sahly, Yulia Laban, Claudia Friedrichs, Torsten Schmidt-Wieland, Klaus Oberdorfer, Roland Pfüller, Thomas Regnath, Corinna Woelke, Carolin Ruckert, Thomas Mertes, Carola Knost-Streicher, Sabine Krämer, Inka Schüttert, Robert Skov, Roman Schwarz, Doris Streit-Schmid, Daniela Walch, Madeleine Mai, Klaus-Peter Hunfeld, Thomas A. Wichelhaus, Andreas Wille, Carolin Muhl, Judith Overhoff

**Affiliations:** 1 Department of Infectious Diseases, Unit for Mycotic and Parasitic Agents and Mycobacteria, Robert Koch Institute, Seestrasse 10, 13353 Berlin, Germany; 2 European Programme for Public Health Microbiology (EUPHEM), European Centre for Disease Prevention and Control (ECDC), Stockholm, Sweden; 3 Department of Infectious Disease Epidemiology, Unit for HIV/AIDS, STI and Blood-borne Infections, Robert Koch-Institute, Berlin, Germany; 4 Department of Infectious Diseases, Postgraduate Training for Applied Epidemiology (PAE), Robert Koch-Institute, Berlin, Germany; 5 Department of Dermatology and Venerology, German Reference Laboratory for Gonococci, Vivantes Hospital Berlin Neukölln, Berlin, Germany; 6 Department of Infectious Diseases, Unit for Sexually Transmitted Bacterial Infections, Robert Koch-Institute, Berlin, Germany

**Keywords:** *Neisseria gonorrhoeae*, EQA, Susceptibility testing, AMR, Antimicrobial resistance

## Abstract

**Background:**

Worldwide, an increase in antimicrobial resistance (AMR) of *Neisseria gonorrhoeae* has been observed. Until now, no protocol for an external quality assessment (EQA) has been available for Germany. The German gonococcal resistance network (GORENET) performed an EQA of primary laboratories in Germany in order to assess quality of antibiotic susceptibility testing, to gain information about laboratory procedures and to assess the impact of these procedures on test results.

**Methods:**

Laboratories assessed drug susceptibility to cefixime, ceftriaxone, azithromycin, penicillin and ciprofloxacin for five *N. gonorrhoeae* strains, using their standard laboratory protocols. Minimal inhibitory concentrations (MICs) were compared to World Health Organisation (WHO) consensus results (or, if not available, reference laboratory results), while deviation by +/− one doubling dilution was accepted. Data on laboratory procedures were collected via a standardised questionnaire. Generalized linear models and conditional inference trees (CTREE) were used to assess relationships between laboratory procedures and testing outcomes.

**Results:**

Twenty-one primary laboratories participated in the EQA in June 2018. 96% of ciprofloxacin MICs were reported within accepted deviations, as well as 88% for cefixime, 85% for ceftriaxone, 79% for penicillin and 70% for azithromycin. The use of interpretation standards and general laboratory procedures like agar base, incubation settings or the use of control strains strongly differed between laboratories. In statistical analysis, incubation time of cultures < 24 h was associated with correct measurements. Additionally, a 5% CO_2_ concentration was associated with correct results regarding azithromycin compared to 3%. CTREE analysis showed that incubation time, humidity and CO_2_ concentration had the greatest influence on the average deviation from consensus results.

**Conclusions:**

In conclusion, we report the development of a protocol for *N. gonorrhoeae* antimicrobial susceptibility testing in Germany. While testing results were in accordance with the expected consensus results in 70–96%, depending on the antibiotic agent, laboratory methodology was heterogeneous and may significantly affect the testing quality. We therefore recommend the development of a standard operating procedure (SOP) for *N. gonorrhoeae* susceptibility testing in Germany.

## Background

*Neisseria gonorrhoeae* is the causative agent of gonorrhea which is estimated to be the third most common sexually transmittable infection globally [1]. Worldwide, resistance to most antibiotics previously available for treatment has been reported, including emerging resistance to extended-spectrum cephalosporins (ESCs) [2]. World Health Organisation (WHO) and the European Centre for Disease Prevention and Control (ECDC) launched a global action plan [1] and a European response plan [3], respectively, in order to control the spread of multi-drug resistant *N. gonorrhoeae* strains. External quality assessment schemes and outcomes have been reported from countries worldwide, including national reports from Australia [4], Canada [5], Latin America and the Caribbean countries [6], India [7] and the WHO Western Pacific and South East Asian Regions [8]. Since 2004, the European Gonococcal Antimicrobial Susceptibility Programme (EURO-GASP) has monitored resistance rates on a European level. Additionally, since 2007, EURO-GASP regularly organized quality assessments for European Reference Centres for Gonococci, in order to ensure high quality and reliability of susceptibility data in Europe [9]. As part of the EURO-GASP network, the German reference laboratory participates annually in the EURO-GASP EQA.

In Germany, gonorrhea is currently not notifiable, which makes estimation of incidences difficult. The German Gonococcal Resistance Network (GORENET) was established in 2013 in order to monitor antimicrobial susceptibility patterns of *N. gonorrhoeae* in Germany. The network recently reported high rates of in vitro resistance for ciprofloxacin, penicillin and azithromycin, while more than 98% of all strains were still susceptible to extended-spectrum cephalosporins (ESCs) [10]. Data acquired within the framework of the project revealed the non-harmonized use of testing systems and interpretation standards between different laboratories. This led to deviations in 36% of the tested samples regarding at least one antibiotic in comparison to results obtained by standardized retesting of isolates at the reference laboratory. Consequently, this altered the susceptibility interpretation in 4% (ceftriaxone) to up to 61% (azithromycin) of samples with deviating measurement results [11]. Therefore, the need for quality assurance of *N. gonorrhoeae* susceptibility testing in Germany became evident.

Until now, no standard operating procedure for antimicrobial susceptibility testing of *N. gonorrhoeae* is available in Germany. While national quality standards in microbiological-infectiological diagnostics (MiQ) are an important source for guidance regarding diagnostics and quality in clinical-microbiological laboratories in Germany, they do not provide detailed procedures for antimicrobial susceptibility testing of *N. gonorrhoeae* [12]. The European committee for antimicrobial susceptibility testing (EUCAST) currently also does not describe a detailed protocol for this cause. Agar dilution for quantitative determination has been replaced by the minimal inhibitory concentration (MIC) gradient strip test methodology in the last years. This is an easier and less time consuming method providing precise and reliable results [13]. However, MIC gradient strip tests are more costly than classical, less accurate test methods such as disc diffusion techniques. Therefore, not all routine laboratories use MIC gradient strip tests. For a few years now, different companies have been offering MIC strip tests for antibiotic susceptibility testing. However, it has been demonstrated that the choice of gradient strip test manufacturers can influence testing outcomes [13], as do bacterial growth conditions, i.e. the choice of media or pH [9].

In our study, we assessed the quality of antimicrobial susceptibility testing for *N. gonorrhoeae* in primary laboratories in Germany and collected data on the laboratory procedures applied. The protocol was developed for routine laboratories in Germany on the basis of the EURO-GASP EQA 2016. In the light of the anticipated mandatory notification for *N. gonorrhoeae* infections in the near future, high quality susceptibility data delivered by primary laboratories are essential for the establishment of a novel surveillance system in Germany. Knowledge on factors influencing the quality of *N. gonorrhoeae* susceptibility testing may also be important for setting up automatized laboratory procedures and development of SOPs.

## Methods

### Antimicrobial susceptibility testing External Quality Assessment (EQA)

Laboratories from the existing pool of twenty-eight GORENET contributors were invited to participate in the EQA [14]. The WHO reference strains [15] used in the EQA were received from the United Kingdom National External Quality Assessment Service (UK NEQAS) in the framework of the EURO-GASP EQA 2016. Five of the strains, WHO U (in duplicate), WHO W, WHO X and WHO Z, were distributed to the participating laboratories on 5th of June 2018. Laboratories were asked to submit results within 3 weeks after receiving the strains. The strains were tested for susceptibility to azithromycin, penicillin, cefixime, ceftriaxone and ciprofloxacin, using the laboratory’s own routine methodologies. Additionally, beta-lactamase testing was performed. For assessment of growth conditions, various commercial manufacturers of incubators are used in the participating laboratories. All devices feature digital electronic temperature, CO_2_- control and integrated humidity control with digital displays. The following information was collected in a standardized online questionnaire (VOXCO Command center 3): susceptibility testing results, interpretation standards used, testing conditions like type of susceptibility testing, culture media, CO_2_ concentration, temperature and humidity settings of the incubator, incubation time, manufacturer/brand of gradient strips, and use of quality control strains. A pilot EQA was performed in April 2017 in order to test time schedule, feasibility and practical procedures. Five selected laboratories participated, testing ten *N. gonorrhoeae* isolates for susceptibility to five antibiotics.

For analysis of performance, consensus minimal inhibitory concentrations (MICs) were defined as the modal MICs described in the Euro-GASP EQA scheme technical report 2016 for azithromycin, cefixime, ceftriaxone and ciprofloxacin [16]. For penicillin, the results obtained in the German reference laboratory were used as reference MIC values. Reported MIC values were compared to consensus and results within +/− 2 values on the gradient strip test scale (equal to +/− 1 one doubling dilution) were accepted as correctly measured. Susceptibility category (SIR) was interpreted according to EUCAST 8.0 breakpoints. Consensus MICs and SIRs are shown in Table S1.

### Statistical analysis of laboratory parameters influencing MIC measurements

Our analysis was based on a subset of six variables which may directly influence the MIC result of the antibiotic susceptibility test, including CO_2_ concentration, incubation temperature, humidity, incubation time, gradient strip manufacturer (for samples measured via this method) and agar base, which were specified as follows: regarding agar bases, the media were distributed into two categories: non-suitable agar bases (selective media) and suitable agar bases (all others). CO_2_ concentration was treated as a binary variable, as only two outcomes were reported. Regarding the variable manufacturer of gradient strip tests, statistical analysis was performed with Etest (bioMerieux, Marcy I’Etoile, France) and MIC test strip (Liofilchem s.r.l, Roseto degli Abruzzi, Italy) measured samples only; these were again treated as binary variables.

To control for the specificity and inherent complexity of the data, a two-step analysis was performed. First, Stata was used to calculate relationships between laboratory/experimental parameters and the outcomes of MIC test results. Separate generalized linear models at both laboratory and antibiotic level were used, assuming a log Gaussian distribution of the outcome [17–19]. In order to omit artificial threshold-effects, the deviation from consensus MIC levels in steps on the MIC gradient strip scale (2 steps = one doubling dilution) was used as outcome variable. Deviations of more than 5 steps were summarized as a single category in order to avoid outliers skewing the data. The laboratory ID was used as random effect to control for unobserved heterogeneity among the individual laboratory characteristics using a log Gaussian linear mixed model framework.

Hereafter, we implemented a nonparametric decision tree algorithm, conditional inference tree (CTREE), to investigate potential dependence structures in the presence of complex and a priori unknown interrelations [20–23]. To this end, a permutation test on independence among covariates (laboratory parameters) and the outcome was performed using the packages party and rpart in R 3.4.1. For the set of covariates with test results (*p*-value) below 0.05, the covariate with the smallest *p*-value was selected as internal node. For this node, the maximum contrast between two samples among each covariate was selected for splitting the tree branches. This selection strategy was continued until no additional significant p-value could be found.

## Results

Twenty-one laboratories participated in the external quality assessment. With one exception, all laboratories submitted the data within the requested three-week period. Twenty laboratories used MIC gradient strips for all five antibiotics included in the susceptibility testing (Table 1). No laboratory used agar dilution for susceptibility testing. Interpretation of the results was performed according to EUCAST in 19 laboratories. Two laboratories interpreted according to the CLSI standard, except for azithromycin for which no CLSI interpretation is available. One of these laboratories used the EUCAST interpretation standard for azithromycin, while the other laboratory did not provide information on the standard used. All participating laboratories reported McFarland 0.5 turbidity for culture inoculation in accordance with EUCAST protocols. In contrast, use of agar base, incubation temperature, CO_2_ concentration and humidity settings of the incubator were heterogeneous (Table 1). Of note, two laboratories used selective agar bases (Martin Lewis agar and *Neisseria* selective agar, respectively) that are not recommended for susceptibility testing. *N. gonorrhoeae* ATCC 49226 was used by 13 laboratories for quality control. Two laboratories used other *N. gonorrhoeae* strains as control (ATCC 19424 and ATCC 43069, respectively), one laboratory did not specify the strains used. Five laboratories did not use any control strains in the course of the susceptibility testing.

**Table 1:** Laboratory specific susceptibility testing parameters and interpretation standards used in participating laboratories

	No. of labs
**Test interpretation according to guidelines**	
EUCAST	19
mixed^a^	2
**Type of susceptibility test used**	
MIC gradient strips	20
MIC gradient strips/disc diffusion^b^	1
Agar dilution^c^	1
**MIC gradient strip manufacturer**	
Biomerieux	4
Liofilchem	10
Oxoid	1
mixed^d^	1
no information provided	5
**Agar base**	
GC chocolatised blood agar	9
Mueller-Hinton chocolatised blood agar	3
Mueller-Hinton fastidious (MH-F)	6
Selective agar	2
No clear information provided	1
**Incubation CO2 concentration**	
3%	2
5%	19
**Incubation temperature**	
35 °C	6
36 °C	13
37 °C	2
**Incubation humidity**	
50%	4
60%	2
70%	4
80%	1
90%	7
100%	3
**Incubation time until MIC reading**	
16 h	1
19 h	1
20 h	5
22 h	2
24 h	12

For azithromycin: EUCAST (1 lab), unknown (1 lab)

^a^CLSI for all antibiotics except for azithromycin

^b^disc diffusion for azithromycin, penicillin, ciprofloxacin

^c^in addition to Etest

^d^Liofilchem exept for ceftriaxone (Biomerieux)

**Fig. 1:**
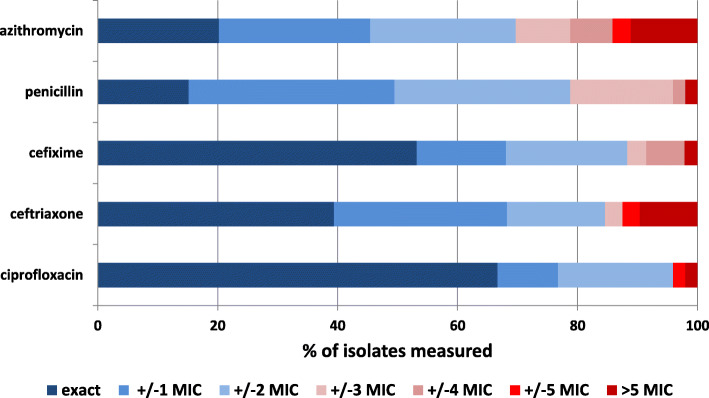
Concordance of minimal inhibitory concentration (MIC) measurement. Distribution of deviation of MIC values from consensus by steps on the MIC gradient strip test scale. Percentage of susceptibility measured isolates per antibiotic over all laboratories are shown. Accepted MICs (within one doubling dilution from consensus) are shown in blue shades. Greater deviations from consensus not accepted as correct measurements are shown in red shades

Four laboratories submitted incomplete susceptibility testing results, either because cefixime susceptibility was not measured (2 laboratories), MIC gradient strips were not used for all measurements and therefore only susceptibility category concordance was reported for these samples (1 laboratory), or measurement results of one sample were not submitted for unknown reasons (1 laboratory). 96% (95/99) of reported ciprofloxacin minimal inhibitory concentrations (MICs) were in concordance with consensus results (Fig. 1). This was the case in 88.3% of cefixime MICs (83/94 samples), 84.6% of ceftriaxone MICs (88/104 samples), 78.8% of penicillin MICs (78/99 samples) and 69.7% of azithromycin MICs (69/99 samples) (Fig. 1).

**Fig. 2:**
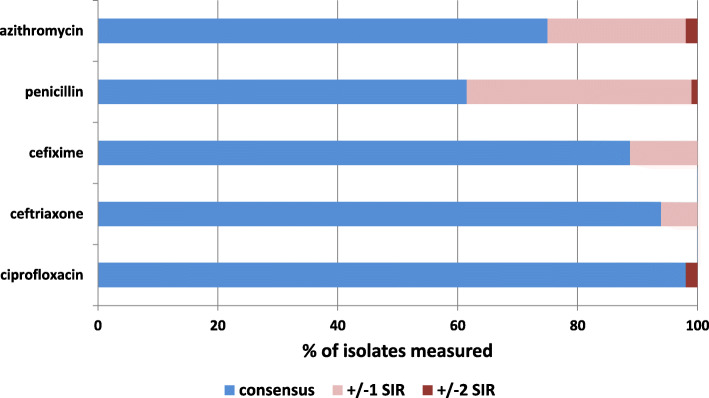
Concordance of susceptibility categorization. Proportion of correctly (blue) and incorrectly (red) categorized samples compared to consensus susceptibility categorization

Regarding susceptibility category, 98.1% of reported ciprofloxacin samples (102/104) were in concordance with the consensus results, while this was the case for 94% (93/99) of ceftriaxone, 88.8% (79/89) of cefixime, 75% (78/104) of azithromycin and 62% (64/104) of penicillin samples (Fig. 2).

For 78/84 samples with incorrectly submitted susceptibility categorization, MIC values were available. Deviations in MIC measurements were responsible for a total of 69% (54/78 samples) of incorrect susceptibility categorizations. Out of these, 54% (29/54) were measured within the accepted range of one doubling dilution. Interpretation of MIC values not in line with the EUCAST scheme was the reason for 28% (22/78) of incorrectly submitted categorizations. For two samples out of these, this was due to interpretation according to CLSI. Both deviations in MIC measurement and incorrect application of the interpretation standard led to incorrect susceptibility categorization in 3% of the submissions (2/78 samples).

Beta-lactamase testing data were submitted by 17 laboratories and 92.9% of the submitted results were in line with consensus results (79/85 samples). Out of the 6 incorrect testing results, 3 were submitted by a single laboratory.

In 15/20 laboratories, the two MIC values submitted for the strain WHO U (distributed as duplicate) were within +/− one doubling dilution (100% intra-lab concordance). For 4/20 laboratories, intra-lab concordance was 80% (4/5 duplicate measurements) and 20% (1/5) for one laboratory. This laboratory additionally failed to report beta-lactamase outcomes in concordance for the duplicate strain. One laboratory did not report MIC values for 3 out of 5 antibiotics and was therefore not evaluated for intra-lab concordance.

The average deviation of MIC values in steps on the gradient strip scale is shown for each laboratory and antibiotic agent in Table S2. Generalized linear model calculation showed that time between growth of the bacterial culture in the presence of the gradient MIC strips and reading of MIC (incubation time, Table 1) significantly influenced average MIC deviation from consensus values for each laboratory. Incubation time less than 24 h was associated with a decreased average MIC deviation by 37% (*p* = 0.003). In detail, each prolongation of incubation time by 1 h was associated with an increase in average MIC deviation by 12% (*p* = 0.000). There was no clear shift to either higher MIC or reduced MIC values with prolonged incubation time. On the antibiotic level, incubation time below 24 h was also associated with the outcome of the first line agent ceftriaxone, with decreased average MIC deviation by 76% (*p* = 0.014).

For azithromycin susceptibility measurements, a 5% CO_2_ concentration during incubation was associated with a decreased average MIC deviation by 54% compared to 3% CO_2_ (*p* = 0.000). Similarly, this could be observed independent of the antibiotic agent (*p* = 0.083). The choice of agar base, incubation temperature, humidity and the choice of the MIC gradient strip manufacturer were not significantly associated with average MIC deviation from consensus values, neither on an individual antibiotic agent level, nor including all measured samples (data not shown).

**Fig. 3:**
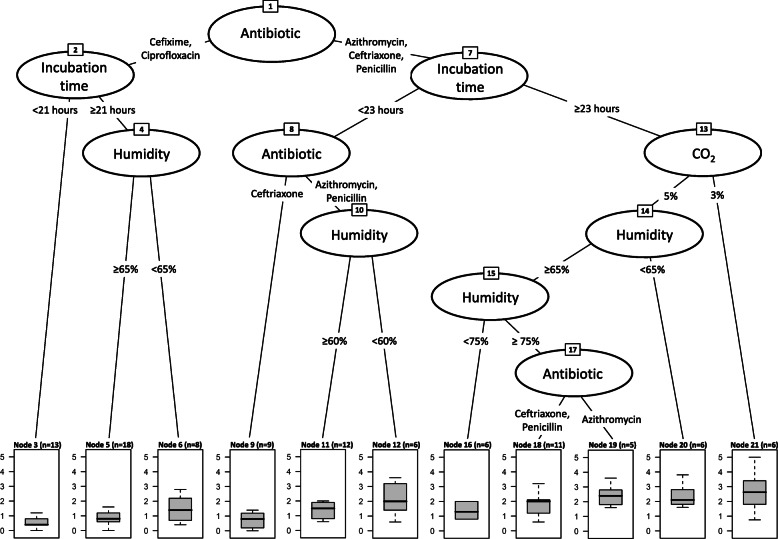
Conditional inference tree model. Laboratory parameters predicted to significantly (*p* > 0.05) influence the testing outcomes. Box plots show median, range and percentiles (25, 75%) of average deviations from the consensus MIC

Our CTREE model consisted of 21 nodes defining 11 homogenous subsets based on four population characteristics (Fig. 3). The first split of data was caused by the covariate defining the antibiotic agent used. Therefore, the influence of further laboratory parameters was strongly dependent on this variable. As shown in the generalized linear model calculation, incubation time and CO_2_ concentration were confirmed as major parameters influencing the outcome of susceptibility testing. Incubation time was important for all samples, but significant data splits happened at different time points (21 h or 23 h), depending on the antibiotic agent measured. In contrast, CO_2_ concentration was only decisive for azithromycin, ceftriaxone and penicillin, if the incubation time was equal to or above 23 h. Humidity was additionally identified as an influencing parameter, which was not significantly associated in the previously described generalized linear model calculation. The influence of humidity settings was complex, causing several data splits in different settings and at different concentrations (60, 65 and 75% humidity) (Fig. 3). The findings of the CTREE calculations were also supported by conditional variable scores (data not shown).

## Discussion

In this study, we report the results of an external quality assessment of *N. gonorrhoeae* susceptibility testing and the influence of heterogeneous laboratory protocols used in routine laboratories in Germany. We were able to identify several laboratory factors which can influence the quality of testing results. Until now, no detailed testing protocol had been developed and EQA was not established for primary laboratories in Germany assessing drug susceptibility of *N. gonorrhoeae*.

Our study design was based on the Euro-GASP EQA 2016, using similar evaluation of MIC concordance and strains [16]. The accepted MIC deviation of one doubling dilution (one 2-fold dilution) was also in agreement with internationally used protocols [4–6]. Considering the pronounced development of resistance in *N. gonorrhoeae* and based on the Euro-GASP EQA 2016, we decided to test five relevant antibiotics (ceftriaxone, cefixime, azithromycin, ciprofloxacin and penicillin). In contrast to the European EQA that included all six WHO reference strains, we decided to use a panel of four strains (5 samples, with one duplicate). Based on feedback from the participating laboratories in the previously conducted pilot EQA, testing of ten samples was not feasible. Therefore, the European Euro-GASP EQA is not entirely comparable with our study. Nevertheless, ciprofloxacin performance was approximately similar (Euro-GASP: 97% MIC concordance; Germany: 96%). European laboratories performed better for cefixime (Euro-GASP: 95%; Germany: 88.3%), ceftriaxone (Euro-GASP: 90.4%; Germany: 84.6%) and azithromycin (Euro-GASP: 84%; Germany: 69.7%), while penicillin was only assessed in our EQA. GORENET laboratories were selected due to their experience in *N. gonorrhoeae* handling and susceptibility testing. However, in contrast to European reference laboratories participating in the Euro-GASP, with long experience in *N. gonorrhoeae* testing protocols, they are no pathogen-specific laboratories. MIC results deviating from consensus values were the reason for the majority of incorrect susceptibility categorizations. As previously reported for Euro-GASP EQA [9], in approximately half of these cases this was due to consensus MIC values close to resistance or susceptibility breakpoints.

The test panel mainly included antibiotics which are currently used for treatment of gonorrhea. According to the 2019 newly issued national guideline for diagnosis and therapy of gonorrhea [24], the calculated treatment of uncomplicated urogenital, pharyngeal or rectal gonorrhea in patients with unknown adherence to therapy and follow-up currently uses a dual therapeutic approach. Ceftriaxone 1–2 g IV or IM in combination with azithromycin 1.5 g PO is recommended as first-line therapy. In compliant patients with reliable follow-up visits (e.g. pregnant women, patients attending special STI clinics), single dose treatment with ceftriaxone 1–2 g IV or IM (as monotherapy) may be given in order to avoid overtreatment and a further increase in resistance to azithromycin. This also applies to patients with a confirmed mono-infection solely caused by *N. gonorrhoeae.* Similar therapeutic recommendations for a reduced use of azithromycin are also given in other countries, partly with complete abandonment of dual therapy [25].

Additionally, there are already known technical issues with susceptibility testing of azithromycin in *N. gonorrhoeae*. Therefore, in 2019, EUCAST indicated an ECOFF (epidemiological cut-off value) instead of standard breakpoints for azithromycin. This new development had not yet taken effect during our EQA.

In the test panel, we included antibiotics which were frequently used for therapy of *N. gonorrhoeae* infections previously, but for which resistance development has been observed. These substances are now being used as second-line or alternative therapeutics. Cefixime in combination with azithromycin can be used as an alternative regime according to the treatment guidelines [24], while ciprofloxacin monotherapy is only recommended if susceptibility was shown in antimicrobial resistance testing.

Penicillin is not part of the recommended standard regimen; however, including the antibiotic into the testing panel allowed for comparison of penicillin susceptibility results and ß-lactamase testing within each laboratory.

As azithromycin and penicillin are less important therapeutically, the relatively poor performance of laboratories regarding testing of these antibiotics might be less important in the future.

Currently, a protocol for *N. gonorrhoeae* antimicrobial susceptibility testing is available from WHO [26] and CLSI [27]; the latter is also recommended by national MiQ recommendations [12]. The European committee on antimicrobial susceptibility testing (EUCAST) however provides separate interpretation standards but does not specify a detailed testing protocol for gonococci (www.eucast.org). Indeed, our analysis showed that most primary laboratories interpreted their results according to EUCAST, but strongly differed regarding their laboratory procedures. Our analysis showed that these differences might impact on the susceptibility testing results.

We identified the incubation time of test strips as a crucial factor influencing susceptibility results. Most laboratories incubated their cultures for 24 h. Indeed, CLSI recommends incubation between 20 and 24 h [27] and this is also stated in manuals of leading gradient strip manufacturers (Biomerieux, Liofilchem). Our analysis showed that incubation time less than 24 h might be beneficial. Interestingly, the EUCAST manual for antimicrobial susceptibility testing via disc diffusion testing recommends incubation between 16 and 20 h for all listed organisms, however not specifying for *N. gonorrhoeae* [28, 29].

We could also show that 3% CO_2_ is associated with less favorable testing results compared to 5%, especially for azithromycin. A CO_2_ concentration of 5% is recommended by CLSI and manufacturer’s manuals. The antimicrobial effect of azithromycin is in fact strongly pH-dependent, but CO_2_ concentrations can also affect cephalosporins by changing the pH of the growth medium [30, 31]. For this reason, the choice of agar base can also be crucial. Most laboratories in our study used GC agar base as advised by CLSI and stated in the protocol of Biomerieux [15] and WHO. Interestingly, several laboratories used MHF including 5% horse blood. Liofilchem specifically recommends MHF agar base for susceptibility testing. EUCAST mentions MHF for fastidious organisms, however not specifying *N. gonorrhoeae*. In fact, a EUCAST publication particularly describes MHF medium as not suitable for *N. gonorrhoeae* [28], making the choice of agar base less clear. The use of chocolatised agar medium in general might be problematic, as the addition of hemoglobin can reduce the result quality [32]. Selective media were used by German laboratories; however, these are important for enriching gonococci from primary samples but are unsuitable for susceptibility testing [9].

The majority of laboratories used *N. gonorrhoeae* ATCC49226 as control strain for susceptibility testing. This strain is recommended as quality control for susceptibility testing. However, other strains representing susceptibility and resistance phenotypes for all tested agents should be used in addition [33]. To this end, we advise primary laboratories to use the isolates provided in this trial and other WHO reference strains on the basis of the WHO global Gonococcal Antimicrobial Surveillance Programme (GASP) recommendations [15].

There are several limitations to our study. First, the statistical power of the study is limited by the number of 21 laboratories. Therefore, the influence of additional laboratory factors on testing results may have not been found in the analysis. For example, we were not able to statistically assess the suitability of agar media. Second, our results might be biased by reporting practices of the laboratories. For example, there could be a tendency to report 24 h incubation time as a standard period, despite possible deviations by laboratories which put less effort into reporting the incubation time accurately, resulting in a worse association of this value in statistical analysis. Third, the participating laboratories were chosen from the existing GORENET network; a mixture of private, university and public laboratories. These laboratories might not necessarily be representative of routine laboratories in Germany handling *N. gonorrhoeae* samples for susceptibility testing, as they are more deeply involved in this technique and therefore part of the network. This might result in a higher quality of testing results compared to laboratories not participating in the GORENET network.

## Conclusions

In conclusion, the study provides evidence that a standard operating procedure (SOP) is needed to facilitate harmonization of laboratory procedures and enhance reliability of testing results for primary laboratories in Germany. Indeed, the GORENET and the reference laboratory for Gonococci in Germany will work together to provide such an SOP in the near future based on WHO, EUCAST and CLSI guidelines, national MiQ recommendations, most recent scientific literature and on the findings of this study. This SOP will guide laboratories regarding all steps of bacterial cultivation, in susceptibility testing with focus on the MIC gradient strip methodology and test interpretation. Additionally, regular training for laboratories will continue to be offered, including recommendations for quality control measures and guidance on handling of strains (collection, storage and transport).

## Supplementary information

**Additional file 1: Table S1.** Consensus values for susceptibility testing. Values for minimal inhibitory concentrations (MIC) and susceptibility categories (R: resistant, I: intermediate, S: sensitive) and outcome of Beta-Lactamase testing used in the study are shown.

**Additional file 2: Table S2.** Absolute average deviation from consensus minimal inhibitory concentration (MIC) for each antibiotic agent tested and laboratory. Steps represent the scale on MIC gradient strips (two steps = one doubling dilution).

## Data Availability

The data sets generated and analysed during the current study are available in the ZENODO (10.5281/zenodo.3333515).

## Abbreviations

AMR: Antimicrobial resistance
ATCC: American Type Culture Collection
CTREE: Conditional inference tree
CL: Consiliary Laboratory for gonococci
CLSI: Clinical and Laboratory Standards Institute
ECDC: European Centre for Disease Prevention and Control
EQA: External quality assessment
ESC: Extended spectrum cephalosporin
EUCAST: European Committee on Antimicrobial Susceptibility Testing
Euro-GASP: European Gonococcal Antimicrobial Surveillance Programme
GORENET: Gonococcal Resistance Network
IM: Intramuscular
IV: Intravenous
MIC: Minimal inhibitory concentration
MiQ: Quality standards in microbiological-infectiological diagnostics
*N. gonorrhoeae*: *Neisseria gonorrhoeae*
PO: Per os (orally)
RKI: Robert Koch Institute
SIR: Susceptibility categorisation (sensitive - intermediate -resistant)
STI: Sexually transmitted infection
SOP: Standard operating procedure
WHO: World Health Organisation
